# Development of an Accelerometer-Linked Online Intervention System to Promote Physical Activity in Adolescents

**DOI:** 10.1371/journal.pone.0128639

**Published:** 2015-05-26

**Authors:** Nicole Guthrie, Andrew Bradlyn, Sharon K. Thompson, Sophia Yen, Jana Haritatos, Fred Dillon, Steve W. Cole

**Affiliations:** 1 HopeLab Foundation, Redwood City, California, United States of America; 2 West Virginia University, Morgantown, West Virginia, United States of America; 3 Santech Inc., La Jolla, California, United States of America; 4 Department of Pediatrics, Division of Adolescent Medicine, Stanford Medical School, Stanford, California, United States of America; Arizona State University, UNITED STATES

## Abstract

Most adolescents do not achieve the recommended levels of moderate-to-vigorous physical activity (MVPA), placing them at increased risk for a diverse array of chronic diseases in adulthood. There is a great need for scalable and effective interventions that can increase MVPA in adolescents. Here we report the results of a measurement validation study and a preliminary proof-of-concept experiment testing the impact of Zamzee, an accelerometer-linked online intervention system that combines proximal performance feedback and incentive motivation features to promote MVPA. In a calibration study that parametrically varied levels of physical activity in 31 12-14 year-old children, the Zamzee activity meter was shown to provide a valid measure of MVPA (sensitivity in detecting MVPA = 85.9%, specificity = 97.5%, and r = .94 correspondence with the benchmark RT3 accelerometer system; all p < .0001). In a subsequent randomized controlled multi-site experiment involving 182 middle school-aged children assessed for MVPA over 6 wks, intent-to-treat analyses found that those who received access to the Zamzee intervention had average MVPA levels 54% greater than those of a passive control group (p < 0.0001) and 68% greater than those of an active control group that received access to a commercially available active videogame (p < .0001). Zamzee’s effects on MVPA did not diminish significantly over the course of the 6-wk study period, and were statistically significant in both females and males, and in normal- vs. high-BMI subgroups. These results provide promising initial indications that combining the Zamzee activity meter with online proximal performance feedback and incentive motivation features can positively impact MVPA levels in adolescents.

## Introduction

Sub-optimal physical activity in youth has been identified as a major public health challenge [1–3]. Recent estimates indicate that only 6–8% of U.S. adolescents achieve the CDC-recommended 60 min of moderate to vigorous physical activity (MVPA) per day [4]. U.S. children’s MVPA levels decline with age, showing a particularly steep drop during middle school years [5,6]. Given the association between low MVPA in adolescents and subsequent low MVPA in adulthood [7–9] and associated elevations in risk for a diverse array of adult chronic illnesses [10–12], there is a great need for effective and efficiently scalable interventions to increase MVPA in middle-school aged children.

Existing interventions to increase physical activity in adolescents range from curriculum-based programs to the use of active video games (i.e., games in which physical movement is part of the gameplay mechanism). Recent literature reviews suggest that no current intervention strategies have been able to consistently induce the sustained changes in MVPA needed to have health benefits in general population samples [13]. However, there is considerable interest in the possibility of improving the physical activity impact of active videogames due to their intrinsic appeal [14], relative ease of distribution, and the potential for such interactive technologies to engage basic positive motivational processes [15,16] that have been identified as key drivers of sustained behavior change [17,18]. Small fixed and intermittent incentives in particular have been shown to promote positive health behaviors in adults [19,20] and may warrant further investigation in the context of adolescent physical activity. In addition, proximal feedback regarding physical activity levels has also been shown to enhance motivation [21,22] and promote sustained behavior change [23,24].

In the present studies, we evaluated a new metrics-driven incentive motivation program called “Zamzee” which seeks to increase MVPA in adolescents by combining proximal feedback on physical activity levels with frequent small scale incentives [25,26]. These two basic principles of behavior change were implemented through the combination of a small wearable 3-axis accelerometer system to assess MVPA (the Zamzee activity meter) and an online feedback and rewards program that seeks to harness basic principles of incentive motivation and intrinsic motivation [17,27]. To maximize efficiency and scaling potential, both components were engineered to be autonomous of program administration staff and as inexpensive as possible while providing accurate MVPA measurement and minimal sufficient external incentives to enhance MVPA over time. This report presents data assessing the performance of the Zamzee activity meter as an accurate measure of MVPA and presents results from a pilot multi-site randomized controlled proof-of-concept experiment testing whether the Zamzee system (activity meter and website) might significantly increase MVPA in middle school-aged children.

## Materials and Methods

### Ethics statement

All study procedures were reviewed and approved by institutional review boards (Ethical & Independent Review Services, Corte Madera, CA, and West Virginia University). Prior to study enrollment, all study participants provided written informed assent in conjunction with parental written informed consent.

### Zamzee activity meter

The Zamzee activity meter is a small, lightweight 3-axis accelerometer system developed by Firefly Design LLC (http://fireflydesign.com/) that implements standard procedures for accelerometer-based activity measurement [28,29] with 10-s data acquisition epochs to ensure detection of the short activity bursts often seen in young people’s spontaneous movement patterns [30,31]. The Zamzee activity meter compiles raw accelerometer output (20 Hz-sampled g values) over 10-s intervals and converts the 3 axial accelerometer measures into a single vector magnitude value (VMa = [(a_x_)^2^ + (a_y_)^2^ + (a_z_)^2^]^.5^, where a_x,_ a_y,_ and a_z_ represent output from each of the 3 orthogonal accelerometers) [28,29]. Based on empirical results from the calibration studies described below, Zamzee VMa values ≥ 4.04 indicate MVPA. VMa values are stored in a flash memory chip with capacity for 12 wks of data, and stored data are automatically uploaded to a web-based data repository whenever the meter is connected to the USB port of a computer running the Zamzee “upload center” software. Separate Zamzee upload centers were developed for computers running Windows (XP, Vista, and 7) and Apple (OSX 10.5 and newer) operating systems.

### Zamzee website

The Zamzee website (previously named gDitty.com) activates automatically when users plug the Zamzee activity meter into the USB port of an internet-connected computer. After the user supplies a login ID and password, their personal website page opens to display two general types of information: 1.) feedback regarding the user’s physical activity history (with a particular focus on the amount of MVPA achieved per day), and 2.) information about the user’s current status within the Zamzee reward system and the nature of the specific rewards available when physical activity performance goals are met. Physical activity feedback is delivered in both a proximal mode, in the form of a graphical time series histogram displaying minute-by-minute activity intensity data over the course of the current calendar day, and in a cumulative summary mode within a separate “Detailed Stats” page presenting numerical measures of total min of MVPA achieved over the past week, total achieved “ever” (i.e., since program initiation), average min of MVPA per day, maximum min of MVPA per day ever achieved, and duration of participation in the Zamzee program, all of which are presented adjacent to a graphical time series histogram displaying daily minutes of MVPA achieved over the past week. Physical activity performance feedback is closely integrated with information about user status within the Zamzee rewards system. The primary Zamee reward system is based on the accumulation of points (MVPA “Zone Activity Minutes”, or ZAMs) that are accrued based on the duration of MVPA achieved per calendar day. ZAMs accrue at the rate of 2 per min of MVPA for the first 20 cumulative min of MVPA per day (a basal incentive level for basic good performance), 8 per min of MVPA for MVPA mins 21–120 (to maximize reward salience for optimal performance), and 0 per min for MVPA mins 121 and above (to avoid incentivizing unhealthy over-activity). Overall reward levels were optimized by balancing 3 competing interests: 1) to ensure that a typical study participant would be able to accrue at least one $5 gift card per week during the course of the study, 2.) to provide sufficient incentive to motivate behavior change in this age group, and, 3) to maximize intervention sustainability by minimizing the financial cost of rewards per unit time. Incentive reward rates were targeted to yield US $5 per wk for a realistic change in MVPA, which was selected to be commensurate with the modal weekly cash allowance provided by parents to middle school-aged children in the US (http://www.kidsmoney.org/allstats.htm). The user’s current accumulated total ZAM balance is displayed numerically adjacent to each activity feedback graph, and daily accrual values are displayed numerically above daily MVPA sums in both daily and weekly performance histograms. Accumulated ZAMs can be redeemed for a variety of tangible monetary rewards (e.g., Amazon.com or Apple iTune gift cards, at $5 value per 1000 ZAMs), or for pro-social rewards such as contributions to a selected charity (e.g., the participant’s school, Save the Children, KABOOM!, or ASPCA, again at $5 per 1000 ZAMs) or self- / value-expressive graphical “badges” that appear on the user’s home page (valued at 250 ZAMs). Reward information and selection buttons are presented adjacent to performance feedback graphs to maximize contingent reward association. In addition to ZAM accumulation based on the continuously accruing sum of MVPA, the reward system also includes a second “level”-based incentive track that specifically rewards achievement of 30 min / d of MVPA (the CDC-recommended level at the time of Zamzee’s initial development) [32,33]. Users progress from one level to another based on the cumulative number of days in which they meet the 30 min /d goal, with gradually increasing numbers of success days required to progress to higher levels. Success in “leveling up” is noted in both text congratulation, display of new achievement level, and presentation of a graphical badge to symbolize each specific level’s accomplishment. To enhance focus on future achievement, text adjacent to the activity performance graphs also indicates the number of additional days of MVPA goal achievement required to reach the next performance level. In addition to activity feedback and reward system information, each user’s home page also displays a user-selected graphical “avatar” character to help promote a sense of self-expression and identity. To harness the performance-motivating effects of social comparison and social facilitation, users also have access to a “People” page that displays the avatar, user ID, and general physical activity performance data (e.g., cumulative sum of MVPA min and current reward system level) for all other active Zamzee system participants in a matrix below the comparable statistics for the user. The overall portfolio of website features was selected to implement basic principles of proximal feedback and performance-contingent reward under a regime that also maximizes intrinsic motivation by enhancing feelings of competence, autonomy, purpose, relatedness, and mastery [17,27]. Access to the intervention website was controlled by an electronic ID programmed into the Zamzee activity meter (i.e., participants in control groups received Zamzee meters that were programmed not to access the Zamzee website, but to instead connect users to a control condition website that simply acknowledged their provision of data to the study).

### Activity meter validation

The Zamzee activity meter was tested for accurate detection of MVPA in a controlled validation experiment in which physical activity levels were experimentally varied across a range from low intensity (e.g., sitting still; 1.1 METs) to high intensity (e.g., sprinting; 10.0 METs) [34] for defined periods of time. 31 middle school-aged children (age 12–14, 17 female and 14 male) were recruited from California middle schools with written parental consent and child assent. Each participant was affixed with a pouch containing the Zamzee accelerometer system (meter) and an established benchmark accelerometer system (RT3; Stayhealthy Inc., Monrovia, CA) [35–39]. Participants were then asked to perform a series of physical activities including sitting at rest (1.1 METs), slow walking (2 mph; 2.0 METs), standing stretches (2.3 METs), normal walking (3 mph; 2.5 METs), brisk walking (4 mph; 3.5 METs), shooting baskets (4.5 METs), jogging (6.0 METs), soccer (7.0 METs), and maximal sprinting (8.0–10.5 METs depending on clocked speed) for experimenter-specified periods of time ranging from 30 s to 10 min. Activities were selected based on 1.) coverage over the range of normal variation in physical activity intensity, and 2.) availability of pre-established metabolic intensity values from previous studies [34].

To validate the technical performance of the Zamzee meter as a measure of physical activity intensity, analyses first quantified relationship between benchmark RT3 activity counts and Zamzee VMa values (VMa = [(a_x_)^2^ + (a_y_)^2^ + (a_z_)^2^]^.5^, where a_x,_ a_y,_ and a_z_ represent output from each of the 3 orthogonal accelerometers). Correspondence between RT3 and Zamzee estimates of activity intensity was quantified by Spearman rank correlation coefficients in both the raw 10 s data epochs in which data were collected and in an aggregated dataset that averaged over repeated measurements on each individual for each activity bout (to control for autocorrelation across repeated epochs from the same person performing the same activity). Ancillary mixed effect linear model analyses were also conducted to relate Zamzee VMa values to criterion RT3 activity counts while controlling for potential correlation of residuals resulting from repeated measurements. Agreement between METs values estimated by Zamzee vs. RT3 was assessed by Bland-Altman plots and related linear regression of agreement (log_10_ Zamzee METs—log_10_ RT3 METs) on average estimated activity intensity ([log_10_ Zamzee METs + log_10_ RT3 METs]/2) as previously described [40]. All analyses were conducted in SAS v9.3 (SAS Institute Inc., Cary, NC), with 2-tailed p < .05 serving as the criterion for statistical significance and a Spearman r > .80 serving as the threshold of acceptable cross-validation.

To identify optimal VMa cut points for identifying MVPA and vigorous physical activity (VPA), receiver operating characteristic (ROC) analyses were conducted in the context of logistic regression analyses relating VMa values to METs values for each activity bout [41,42]. Analyses were conducted using SAS PROC LOGISTIC to identify a threshold VMa value below which the Zamzee meter was assumed to be perfectly stationary (i.e., not actually worn by a person), 2) a threshold VMa value corresponding to the 4.0 MET threshold of MVPA conventionally used in studies of energy expenditure in children [3,41,42] a threshold VMa value corresponding to the conventional 6.0 MET threshold of VPA. Ancillary Spearman correlation and mixed effect linear model analyses were also performed to gauge the magnitude of monotonic relationship between VMa values and METs over the entire range of activity intensities examined.

### Pilot study of effects on physical activity

To test for potential impact of the Zamzee meter/website system on MVPA levels in 11–14 year-old children, we conducted a 6-week multi-site randomized controlled pilot experiment (ClinicalTrials.gov Identifier: NCT02425384) involving 3 study arms: 1) a passive control group that received Zamzee activity meters but no other intervention (i.e., had no access to the Zamzee website), 2) an active control group that received Zamzee activity meters and the Dance Dance Revolution (DDR) active video game (Konami Inc., El Segundo CA), which has previously been shown to increase MVPA [43,44], reduce sedentary time [45] and improve health risk factors [46] in youth, and 3) a Zamzee intervention group that received Zamzee activity meters that were programmed to access the Zamzee feedback and incentive motivation website. Participants were a convenience sample recruited by community flyers and in-person presentations at middle schools in Morgantown WV, Mountain View CA, and Vista CA. Enrollment criteria were 11–14 yrs of age, ability to read English, and ability to participate in normal physical activity. Criteria were ascertained by self-report and signature on the informed consent form. Participants were enrolled following parental written informed consent, dispensed a Zamzee activity meter, and randomized to experimental conditions using a random number generator with 1/3 probability of assignment to each condition.

In all study arms, participants were asked to wear their Zamzee activity meters close to the trunk of their bodies (e.g. clipped to their waistband or in their pants pocket) during all waking hours (except when in water). Participants were asked to charge the meter periodically (every couple of days) using a provided AC charger or by connecting it to a computer USB port. To facilitate activity data collection, participants were asked to periodically connect their Zamzee activity meters to the USB port of an internet-enabled computer (e.g., at home or school) on which Zamzee upload center software had been installed.

Passive and active control participants saw a study participation acknowledgement webpage when they initially uploaded data, but saw no information about their physical activity levels (no feedback) and had no access to any other intervention stimuli (no rewards or other incentives). Active control group participants received a version of DDR (including dance mat and instruction manual) specific to whatever game console they reported to have available at home in a pre-study survey (Sony Play Station, Windows PC/Xbox). Participants who did not own a DDR-compatible computer or videogame console were loaned a Play Station console (Sony Computer Entertainment America LLC, Foster City CA) to support DDR use for the length of the study. The DDR dance mats were equipped with tracking software to record the date, time, and duration of each DDR game play session. Participants were asked to use the assigned program (Zamzee website or DDR game) as little or as much as they wanted to over the course of the 6-wk study.

The primary outcome analyzed was average daily MVPA levels aggregated weekly over the 6-wk study. Daily MVPA was quantified as both absolute duration of MVPA per day (i.e., sum of 10 s VMa values per day) and as the MVPA rate, or the ratio of MVPA duration to the total duration of time monitored per day (i.e., sum of 10 s VMa values exceeding the MVPA threshold divided by the sum of 10 s VMa values exceeding the “meter worn” threshold of .007). Data were analyzed by mixed effect linear models (SAS PROC MIXED) using a 3 (Group) x 6 (Time) factorial design. In the event of a significant omnibus test statistic for the overall Group main effect or the Group X Time interaction, follow-up contrast analyses assessed the statistical significance of pairwise differences among groups. Analyses were conducted on an intent-to-treat basis, with all available data analyzed for any day in which VMa values exceeded the “meter worn” threshold for > 10 min. Days in which VMa exceeded the “meter worn” threshold for < 10 min were treated as missing data (i.e., assumed to represent spurious readings rather than valid assessment of daily activity). To ensure that individual differences in meter wear times did not bias results, analyses of MVPA duration controlled for individual differences in meter wear time as a covariate, and analyses of MVPA rates (i.e., MVPA duration / total wear duration) were weighted by daily meter wear duration. All analyses also controlled for study site to address potential clustering. In addition to primary analyses of general experimental group effects, ancillary analyses tested for potential differences in the magnitude of experimental effects as a function of participant sex (self-reported at study entry) and adiposity (determined by research staff measurement of height and weight at study entry). BMI was computed based on measured height and weight, converted to age- and sex-specific percentile values, and classified as normal BMI for values < 95% or high BMI for percentiles ≥ 95% (the CDC-recommended cut-off for youth BMI classification) [47]. In the absence of any prior information regarding the Zamzee system’s likely effect on MVPA levels, this study was powered to detect a moderate effect size (.5 SD) with > 90% power in a planned contrast comparing the 6-wk average MVPA level in the Zamzee group with the pooled 6-wk average observed in the active and passive control groups.

At study completion, all participants received compensation deemed appropriate by institutional review boards in consultation with local site administrators (Site 1, Morgantown WV: $50 gift card to participants and $25 gas card to parents to compensate for transportation requirements within the broader WV region from which participants were drawn; Site 2, Mountain View CA: $60 cash; Site 3, Vista CA: $30 cash). On average, Zamzee participants received an additional $7.55 in non-cash activity rewards (gift cards, charitable donations made in their name, etc.).

## Results

### Activity meter calibration

**Fig 1:**
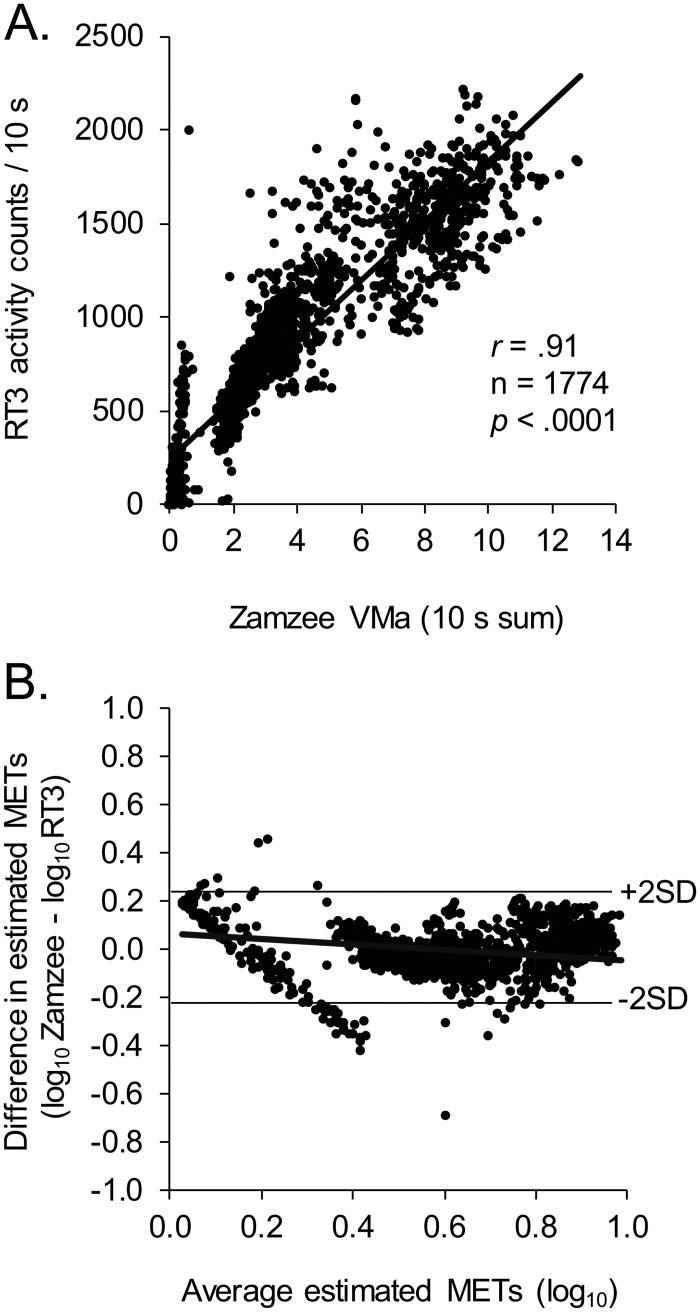
Correspondence between Zamzee and RT3 activity meters. Physical activity was experimentally varied by asking 31 middle school-aged adolescents (12–14 years of age) to sit at rest, perform standing stretches, walk slowly, walk normally, walk rapidly, shoot baskets, jog, play soccer, and sprint for .5–10 min intervals while wearing both RT3 and Zamzee activity monitors. (A) Zamzee activity measurement (3-d accelerometer vector magnitude; VMa) and RT3 activity counts were accumulated over 10 s intervals, and the magnitude of their monotonic relationship was assessed by Spearman r correlation coefficients. Solid line indicates linear regression function. Similar results emerged from analyzes of data that averaged over repeated measurements within individuals to form a single summary mean for each activity (n = 96, r = .97, p < .0001). (B) Bland-Altman plot of agreement between MET values estimated by Zamzee vs. RT3 (difference: log_10_ Zamzee—log_10_ RT3) as a function of MET intensity (average: [log_10_ Zamzee + log_10_ RT3] / 2). Solid line indicates linear regression function.

Fig 1 shows the correspondence of measurements from the Zamzee activity meter compared to the benchmark RT3 activity meter over 1,774 10 s epochs of experimentally manipulated physical activities that included seated rest, performing standing stretches, slow walking, normal walking, fast walking, jogging, shooting baskets, playing soccer, and sprinting. Data were collected from 31 middle school-aged children (12–14 years of age, 55% female). Results show a close monotonic correspondence between Zamzee VMa values and RT3 activity counts, with a Spearman correlation of r = .94 (p < 0.001; Fig 1a). Similar results emerged when analyses averaged over repeated 10 s epochs to form a single average VMa value for each person during each activity (n = 96, Spearman r = .97, p < .0001). MET values estimated by Zamzee showed close agreement with those estimated by RT3, as shown by Bland-Altman plot (Fig 1b) indicating absence of systematic bias (mean difference between Zamzee- and RT3-estimated METs = 0.7% of their average value) and no trend toward greater % disagreement at greater METs intensity (minimal/negative slope of regression line in Fig 1b).

**Fig 2:**
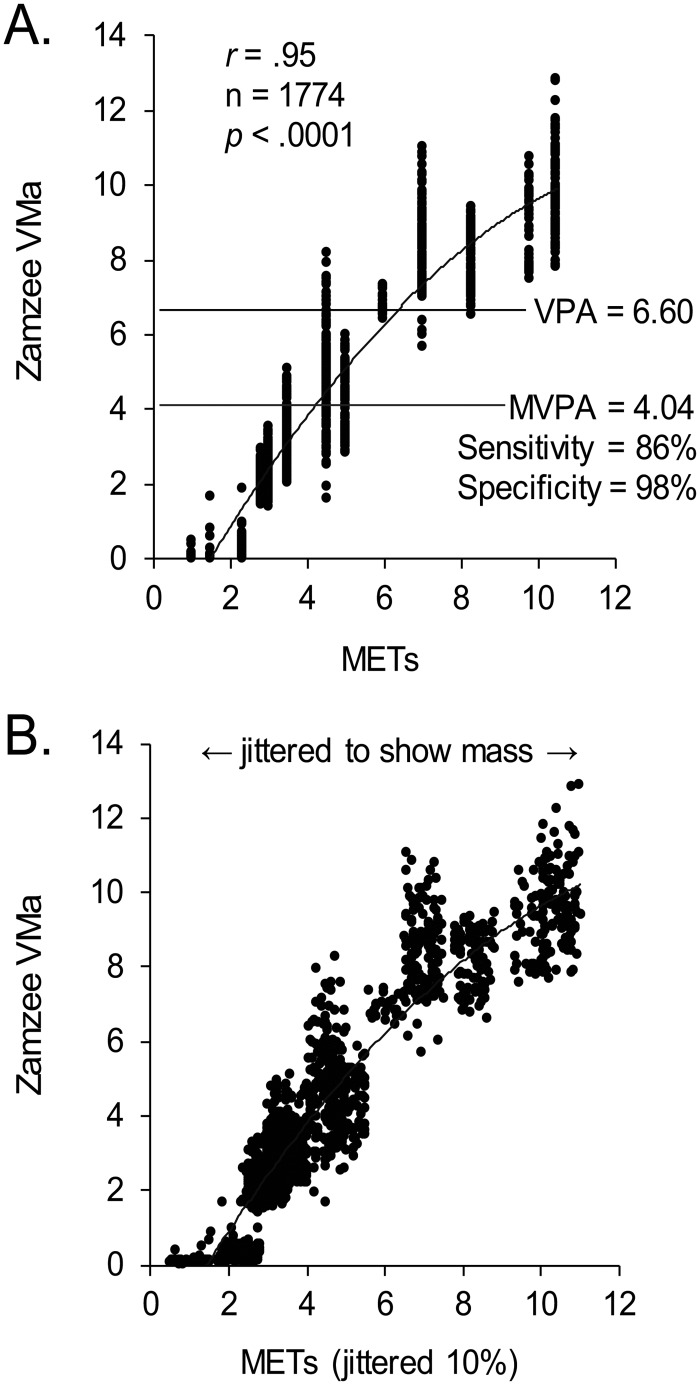
Correspondence between Zamzee VMa values and experimentally manipulated activity intensity (in METs). (A) Receiver operating characteristic analyses were applied to the parametric physical activity calibration data (described in Fig 1) to identify optimal accelerometer VMa cut points for distinguishing moderate-to-vigorous physical activity (MVPA = activity ≥ 4 MET intensity) and vigorous physical activity (VPA = activity ≥ 6 MET intensity). r and p values derive from ancillary Spearman rank correlation analyses to assess monotonic relationship between VMa and METs over the entire range of activity intensities examined.

Fig 2 shows results from receiver operating characteristic (ROC) analyses applied to the same set of parametric calibration data to identify optimal cut points for distinguishing MVPA and VPA. Results identified a VMa value of 4.04 as the optimal threshold for detecting MVPA (METs ≥ 4), yielding a sensitivity of 85.9%, a specificity of 97.5%, and a 97.5% total concordance between VMa and METs (p < .0001). A VMa value of 6.60 was identified as the optimal threshold for detecting VPA (METs ≥ 6; sensitivity = 98.0%, specificity = 98.8%, concordance = 99.8%; p < .0001). These results compared favorably with results for the benchmark RT3 accelerometers (MVPA: sensitivity = 86.7%, specificity = 95.7%, concordance = 97.5%; VPA: sensitivity = 77.7%, specificity = 95.0%, concordance = 95.5%). MVPA duration was thus estimated in subsequent studies as the sum of 10 s monitoring epochs in which the Zamzee VMa value exceeded 4.04.

### Pilot study: effect of Zamzee on physical activity

To assess the Zamzee system’s potential impact on MVPA in adolescents, 182 middle school-aged children were recruited from 3 study sites and randomized into a passive control condition (no intervention; n = 59), an active control condition (DDR active videogame; n = 61), or an intervention group that received access to the Zamzee online incentive rewards system (n = 62). Total and site-specific sample characteristics are presented in Table 1.

**Table 1:** Sample Characteristics.

	ALL	Passive Control	Active Control (DDR)	Zamzee
**n**	**182**	**59**	**61**	**62**
**Age,** mean (SD)	12.7 (0.9)	12.5 (0.8)	12.7 (0.9)	12.7 (0.9)
**BMI,** mean (SD)	21.2 (4.6)	22.2 (5.1)	20.7 (3.9)	20.7 (4.5)
**Female,** n (%)	88 (48)	23 (39)	31 (51)	34 (55)
**Ethnicity,** n (%)				
** Caucasian**	97 (53)	30 (51)	32 (52)	35 (56)
** Black or African American**	21 (12)	4 (7)	9 (15)	8 (13)
** Asian**	10 (5)	3 (5)	2 (3)	5 (8)
** Hispanic**	37 (20)	16 (27)	12 (20)	9 (15)
** Other/ Decline to answer**	17 (9)	6 (10)	6 (10)	5 (8)

#### Adherence and intervention utilization

Study protocol adherence was high, with 96% (175/182) of participants providing physical activity data over the course of the 6-wk study. On average, participants provided valid activity data on 63% of the study days (mean = 26.4 ± SE 13.2 days) and wore their activity meters for 8.9 ± 0.1 hrs / day. Measurement non-adherence rates were similar across study groups: participants who provided no activity data included n = 3 in the intervention group, n = 3 in the passive control group, and n = 1 in the active control group. In the Zamzee intervention group, 82% of participants visited the intervention website at least once. In the active control group, electronic tracking of DDR dance pad activity indicated that 51% of participants used the assigned game at least once. Intervention group participants earned a median US $5.00 / wk in incentive rewards (25^th^ percentile = $1.60; 75^th^ percentile = $8.30).

#### Primary outcome: moderate-to-vigorous physical activity

**Fig 3:**
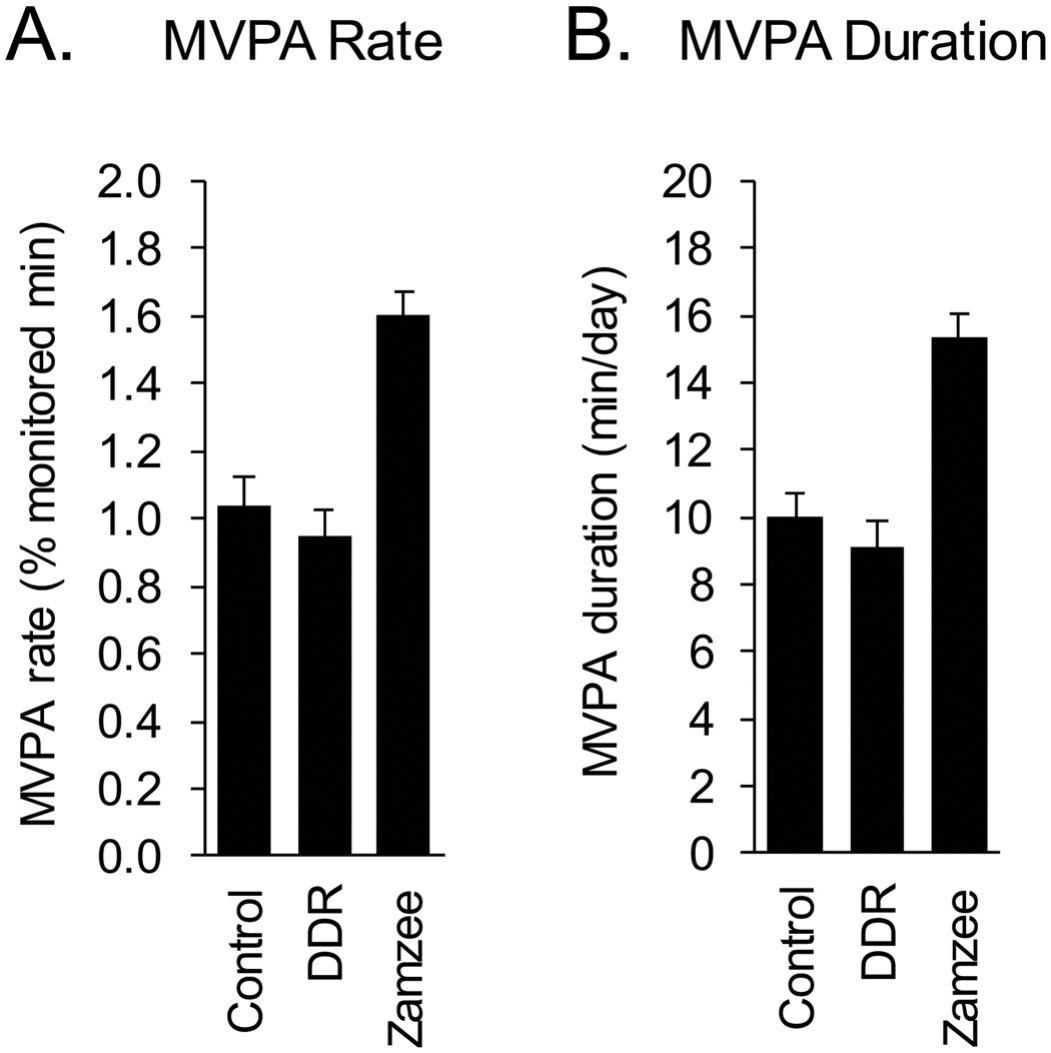
Moderate-to-vigorous physical activity (MVPA) across experimental conditions. Average (±SE) daily MVPA rate (MVPA duration as a fraction of total time monitored) (A) and standardized MVPA duration (i.e., standardized to a 16 h waking day) (B) in passive control condition participants, active control participants who received a DDR active video game, and intervention group participants who received access to the Zamzee online feedback and rewards system.

Weekly average MVPA duration (min/day) and MVPA rate (min/monitored duration) were analyzed in a 3 (Group) x 6 (Week) mixed effect linear model, with planned contrasts assessing pair-wise differences between groups in average MVPA over the entire 6-wk period. As shown in Fig 3, average MVPA duration differed significantly across groups (p < .0001). Participants in the passive control group showed an average 10.27 min of MVPA per day over the course of the 6-wk study and participants in the active control group (DDR) showed an average 9.12 min MVPA per day. Average MVPA duration did not differ between active and passive control conditions (p = .2732). Participants in the Zamzee intervention group showed an average 15.26 min of MVPA per day—a level 49% greater than that of the passive control group (p < .0001) and 67% greater than that of the active control group (p < .0001). The magnitude of Zamzee intervention effects did not differ significantly over the course of the 6-week study (i.e., no significant Group x Week interaction; p = .6048). (Fig 3a shows observed differences in directly measured MVPA rates and 3b expresses the same data normalized to a standard 16 h waking day.)

#### Sex differences in intervention impact

To determine whether Zamzee intervention effects might differ for females vs. males, ancillary analyses were conducted in a 2 (Sex) x 3 (Group) x 6 (Week) factorial design. Forty-eight percent of participants were female, and the prevalence of female participants was similar across experimental conditions (n = 23 in passive control, n = 31 in active control, and n = 34 in Zamzee intervention group; difference p = .1952 by *X* ^2^). Results showed 38% greater MVPA levels in general for males vs. females (p < .0001), as well as a continued significant effect of Zamzee in promoting overall MVPA rates (p < .0001). Results also identified a statistically significant sex difference in the magnitude of Zamzee intervention effects (p = .0384), with female Zamzee participants showing an average 39% increase in MVPA (p = .0049), and male Zamzee participants showing an average 76% increase in MVPA (p < .0001).

#### Effect of adiposity on intervention impact

To determine whether Zamzee intervention effects might differ for individuals who were overweight at study entry, additional ancillary analyses were conducted in a 2 (BMI: normal vs. high) x 3 (Group) x 6 (Week) factorial design. Sixty-four percent of participants had normal BMI-for-age/gender percentiles (i.e., < 95^th^ percentile of CDC reference norm), and the prevalence of high-BMI participants was similar across experimental conditions (n = 15 in passive control, n = 11 in active control, and n = 10 in Zamzee intervention group; difference, p = .4020 by *X* ^2^). Results identified no significant BMI x Group interaction for MVPA (p = .9146), and Zamzee intervention effects on MVPA remained highly significant in analyses that included BMI group as a factor (p < .0001).

## Discussion

These results indicate that the Zamzee activity meter provides a valid measure of moderate-to-vigorous physical activity (MVPA), and that the Zamzee system (activity meter with an online incentive motivation system) can increase MVPA rates in 11–14 year-old children by > 50% over a 6-wk period. Zamzee intervention effects on MVPA did not diminish significantly over the course of the 6-wk study period, and were statistically significant in both males and females and in both normal- and high-BMI subgroups. These findings provide a promising proof-of-concept indication that the Zamzee system’s combination of comprehensive activity monitoring, online feedback, and incentive motivation can increase MVPA in a community sample of middle school-aged American youth.

The present studies were intended to provide a “real world” assessment of the Zamzee system’s performance. Although intervention study participants were not required to use the Zamzee website, > 80% of participants given access to the Zamzee website chose to use it (a rate notably greater than the 51% who chose to use the DDR active video game utilized in the active control condition). The Zamzee activity monitor also appears to be a relatively acceptable device for measuring physical activity in adolescents, as indicated by the fact that valid activity data were obtained for more than 60% of total study days (despite the fact that participants were only verbally requested to wear their Zamzee meters and no other actions were taken enforce this request over the course of the 6-wk study). Previous studies using technology-based interventions to promote physical activity have found that maintaining user engagement can be a significant challenge [46,48]. The present engagement results suggest that the Zamzee system may provide a promising new tool for promoting physical activity in children. The relatively low price-point of the Zamzee system (approximately $30 meter + $5/wk rewards in its current configuration) and the internet-based deployment of the Zamzee incentive motivation system should also help facilitate the scaling and distribution of interventions based on this platform.

Our results suggest that the Zamzee system may provide an effective system for promoting MVPA in adolescents, but the specific psychological mechanisms mediating the observed effects remain to be identified in future research. Provision of website-mediated financial rewards (in the form of gift cards) seems likely to have played a significant role in these effects, as previous studies have shown similar impacts of financial incentives on other health behaviors such as medication adherence, smoking cessation, and weight loss [19,20,49]. However, the Zamzee website was designed specifically to maximize intrinsic motivation by combining minimal sufficient tangible rewards with an array of non-financial incentives such as regular indications of personal progress, social comparison and facilitation components, self-determination of rewards and the specific activities undertaken to achieve them, and the ability to support charitable causes. Previous research on physical activity motivation suggests that simply providing feedback (e.g., in the form of pedometer metrics) can motivate changes in behavior [21,22]. To maximize self-determination and sense of control (key psychological ingredients of intrinsic motivation [17], the Zamzee system was also designed to provide users maximal flexibility in selecting the specific types of physical activity they might engage in to acquire MVPA ZAMs. The more salient presentation of rewards (both intrinsic and extrinsic) and the greater flexibility in achieving activity goals might potentially explain why Zamzee outperformed the more structured and focused DDR active video game in promoting MVPA in the present study. Future research involving parametric variation of website features, extrinsic reward rates, and other content will be required to more precisely define the psychological mechanisms of Zamzee’s observed effects on physical activity. Although the current incentive system was clearly sufficient to incentivize behavior change in this middle school-aged population, further optimization of reward levels and rates could potentially yield additional increments to MVPA.

The present meter validation studies and randomized experimental results are promising, but these findings remain preliminary and are limited in several respects. The meter validation studies involved brief experimental manipulations of physical activity intensity in relatively small samples. Future validation studies using additional physical activity exposures (including extended observation of naturally occurring variations in activity) [30] in larger and more diverse samples are warranted. The present randomized controlled pilot experiment provides some indication that the Zamzee system might positively impact MVPA in adolescents, but future studies examining MVPA levels over longer durations (e.g., 6–12 mo) and in larger samples are required to verify these initial findings. Larger samples would provide greater statistical power to assess potential differences in Zamzee intervention effects across demographic groups (e.g., sex, race/ethnicity, socio-economic status, older and younger age groups) and across behaviorally-defined risk groups (e.g., low-activity or overweight/obese sub-populations). Although promising in direction and statistical significance, the magnitude of Zamzee effects on MVPA in the pilot study was not large in absolute terms (approximately 5 min/d, or a Cohen’s d = .4 SD). The average increment to MVPA observed here would mitigate approximately 23%-34% of the net 110–165 kcal/d energy gap estimated for current U.S. adolescents [50] (assuming a 50 kg person running at 6 mph as MVPA). As such, these results should be treated as initial proof-of-concept data to motivate further optimization of the Zamzee intervention system to enhance its impact. Moreover, the present study provides no information about the persistence of MVPA differences following study termination and no measures of their potential health impact (e.g., no assessments of change in BMI or biomarkers of the underlying physiologic processes that mediate effects of a low-MVPA lifestyle on disease risks). As noted above, the Zamzee online incentive motivation system sought to tap multiple psychological processes to help motivate MVPA and minimize the adverse effects of extrinsic motivation [17,27]. Due to Zamzee system administration of rewards with monetary value (i.e., gift cards and charitable contributions), participants in the Zamzee condition received greater total compensation than did participants in the control condition. This provides a valid test of the concept of incentivizing desired MVPA, but it also complicates the interpretation of differences between groups and leaves open the question of whether similar effects could be obtained without such monetary incentives. Future parametric studies will be required to assess the relative contributions of the multiple motivational features involved in the present system and gauge the potential of additional features to enhance its impact.

Despite the early stage of the Zamzee system’s development, and the limited data available so far, the present studies do suggest that the Zamzee activity meter can provide a valid and engaging instrument for measuring MVPA in adolescents, and that integrating this meter with an online feedback and incentive motivation system can significantly increase MVPA levels over a 6-wk period in a community sample of normal adolescents. Given the public health significance of maintaining healthy levels of physical activity in young people [1–3] and the relative difficulty of significantly increasing adolescent MVPA in previous studies [13,46,48], these initial proof-of-concept results may provide a platform for future research to enhance the impact of the present Zamzee system and provide more comprehensive and robust assessment of its impact on physical activity and health.

## Supporting Information

S1 File Zamzee system website content. (DOC)

S1 Dataset Activity meter calibration study data. (XLSX)

S2 Dataset Pilot study data. (XLSX)
