# Supplementary material for: Development of an Accelerometer-Linked Online Intervention System to Promote Physical Activity in Adolescents
Source: PLoS One. 2015 May 26;10(5):e0128639. doi: 10.1371/journal.pone.0128639 (PMC4444279; doi:10.1371/journal.pone.0128639)

**S1 File – Zamzee intervention website images**

Note: At the time this research was performed, the Zamzee intervention system was code-named “gDitty” (which thus appears on many of the following webpages).

Image 1 - Website login page.


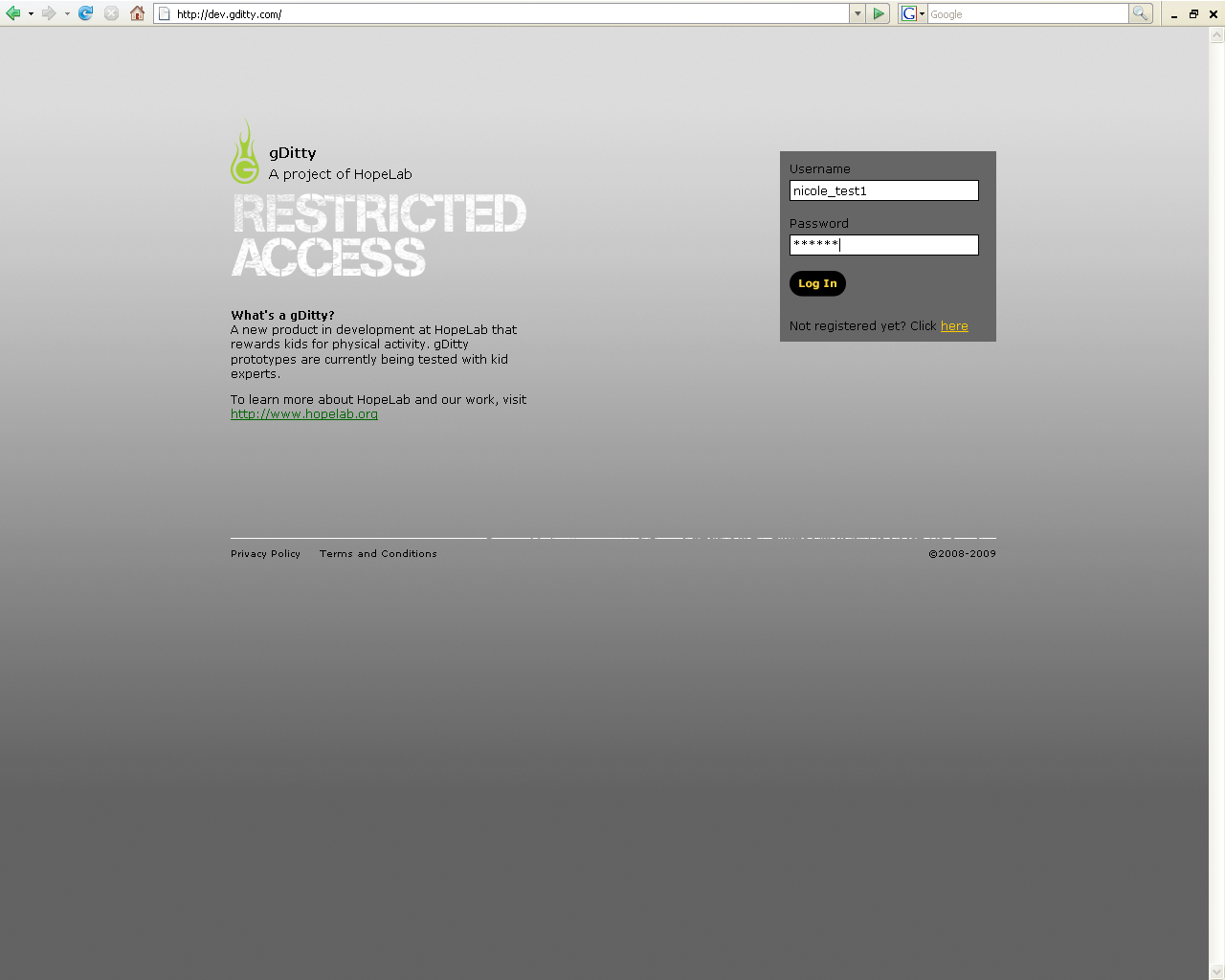


Image 2 – Individual profile page: current day view


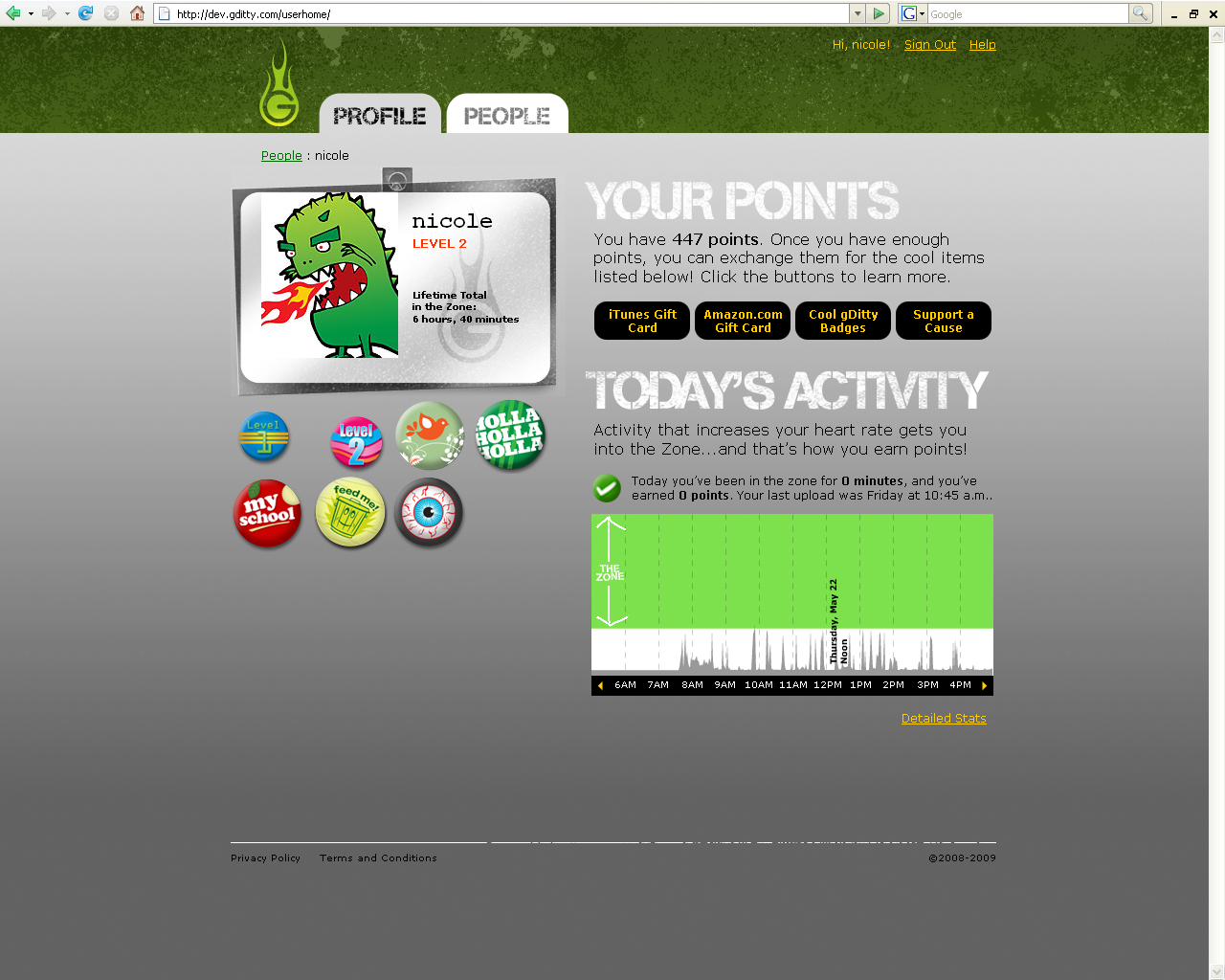


Green activity plot:

Horizontal axis = time of day

Vertical axis = activity intensity during specified period of time (green “zone” = MVPA)

Image 3 – Individual profile page: current week view


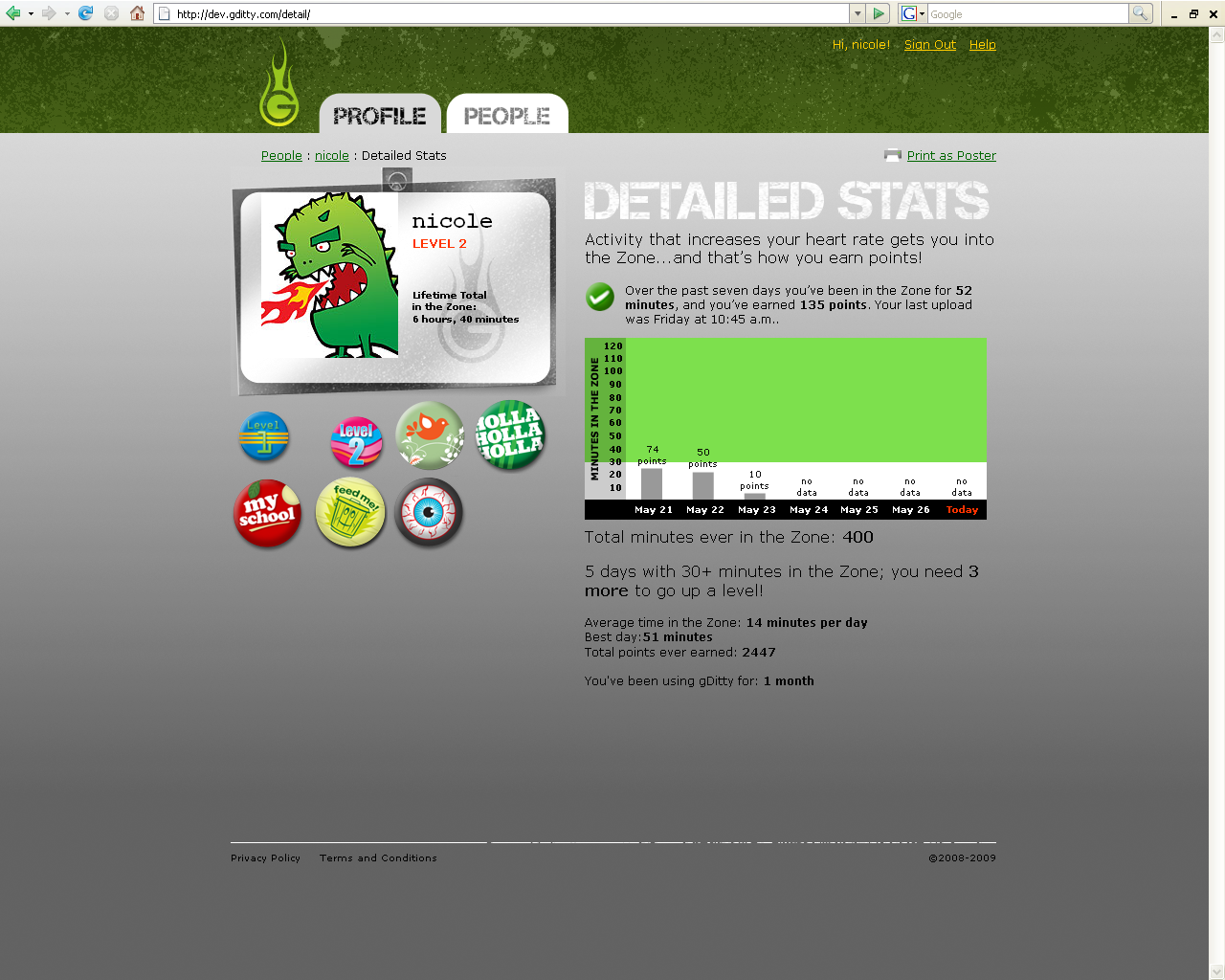


Green activity plot:

Horizontal axis = day of week

Vertical axis = duration (min) of MVPA (“in the zone”) per day

Image 4 – Individual profile page: incentive badge options


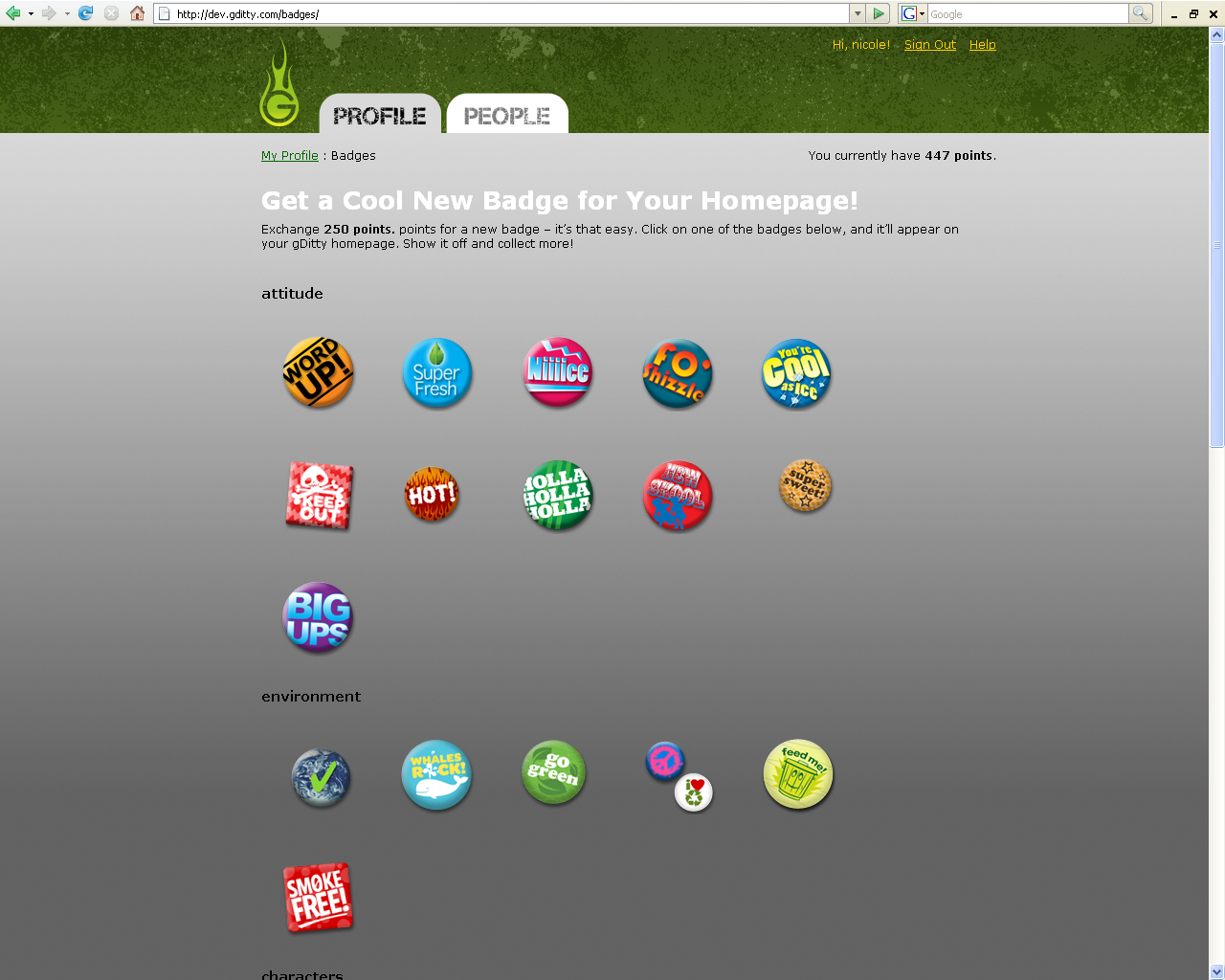


Image 5 – Individual profile page: new incentive badge awarded


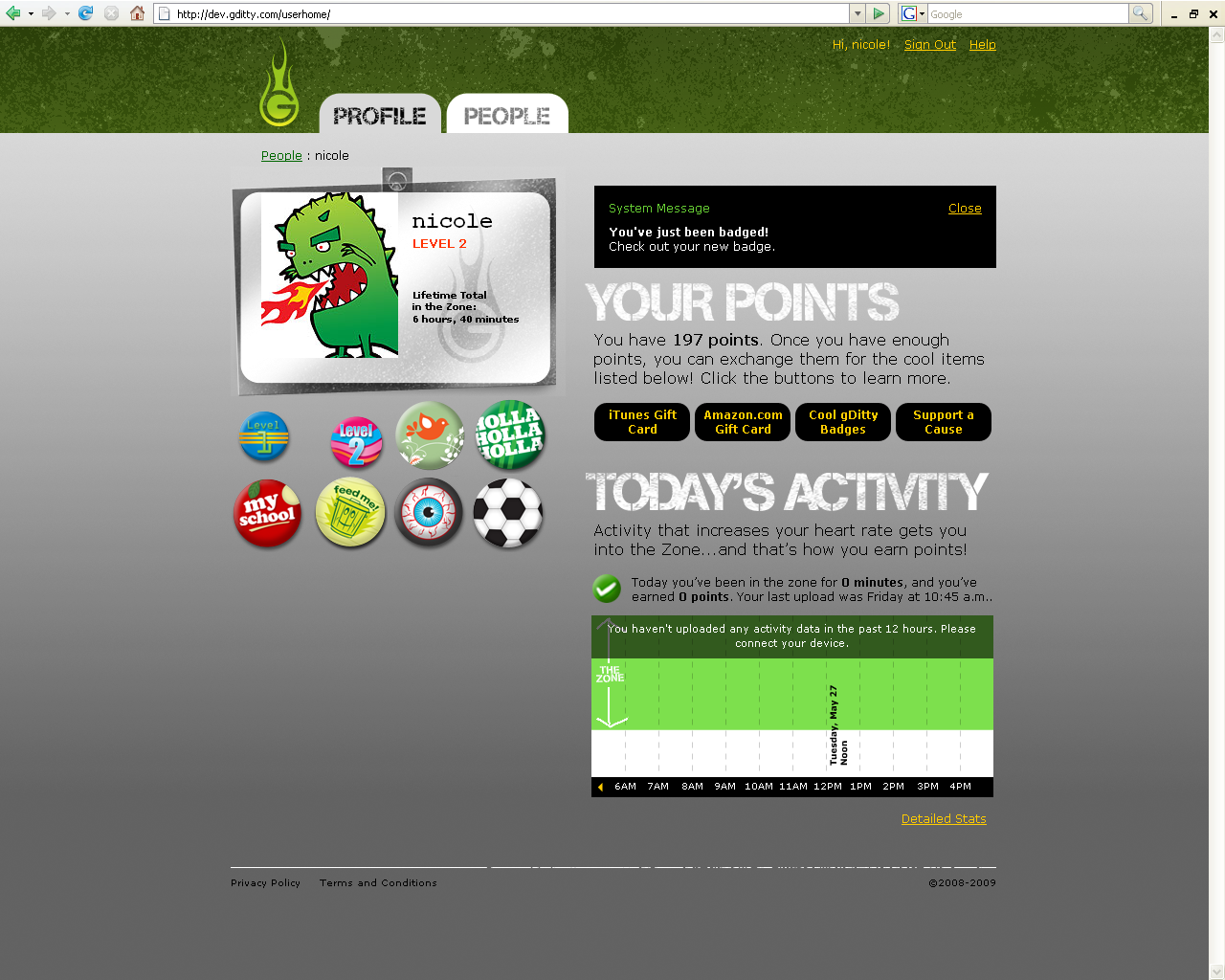


Image 6 – Individual profile page: incentive charitable donation options


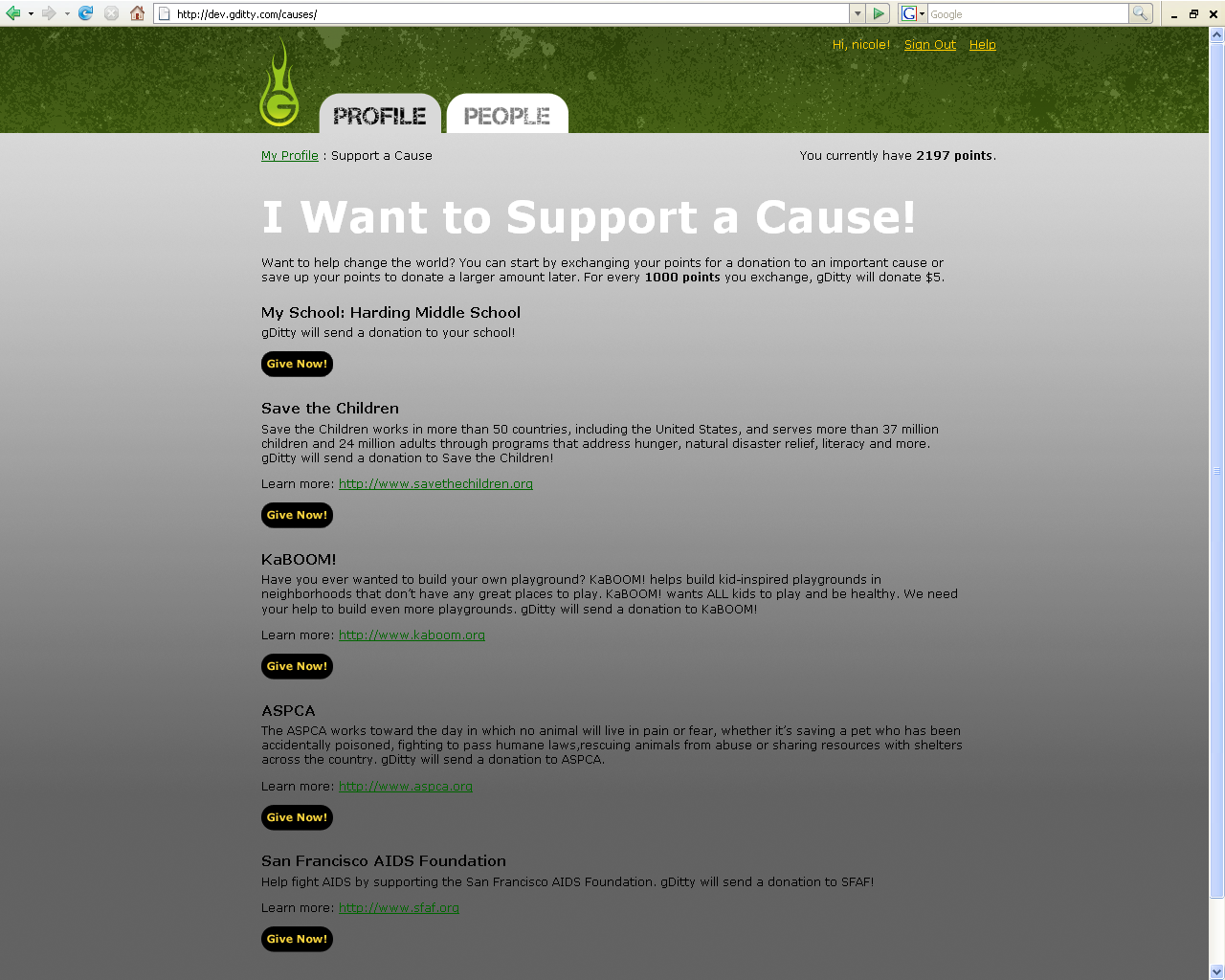


Image 7 – Individual profile page: incentive charitable donation to participant’s school


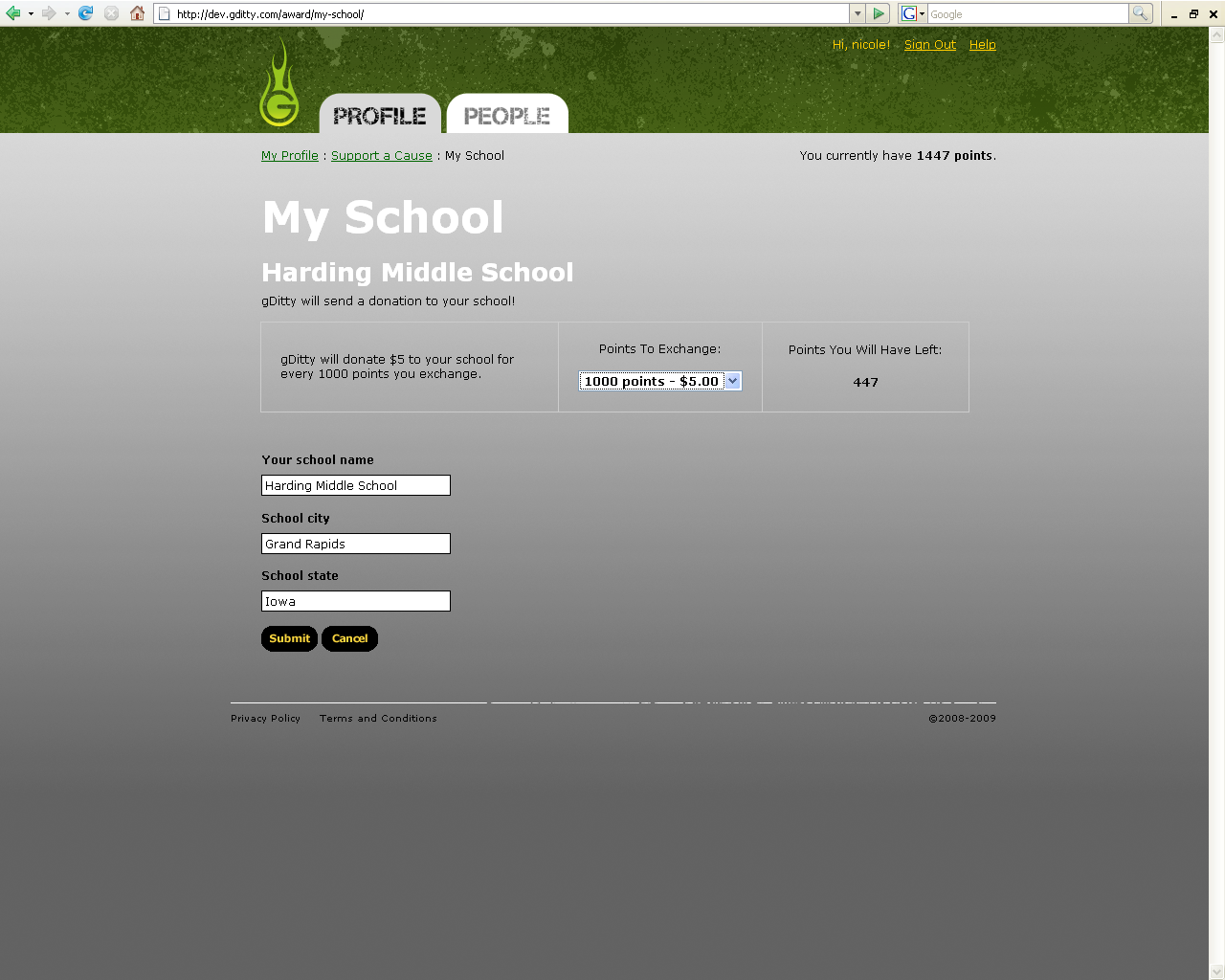


Image 8 – Individual profile page: badges noting incentive charitable donation to participant’s school


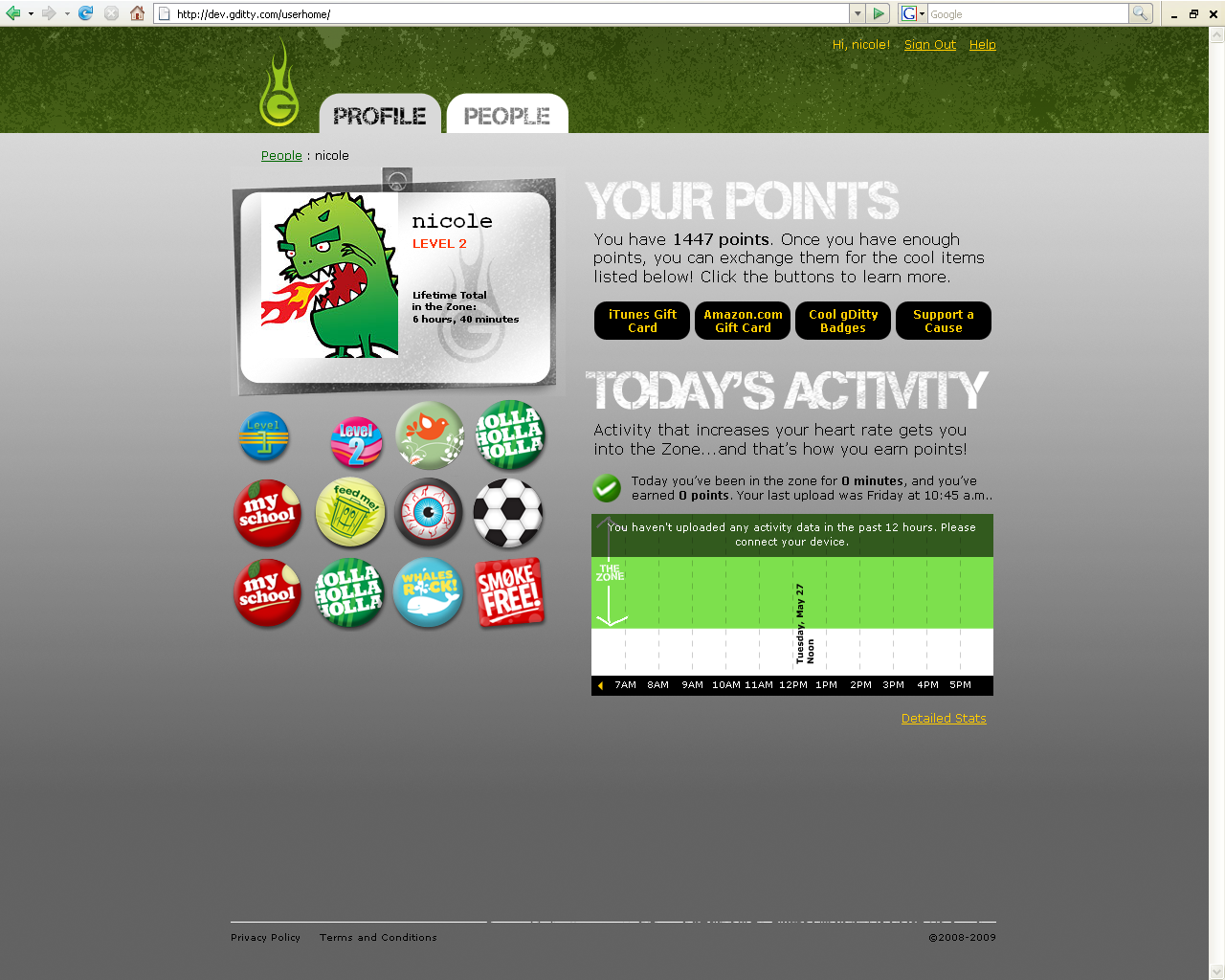


Image 9 – Individual profile page: converting activity points to incentive gift card


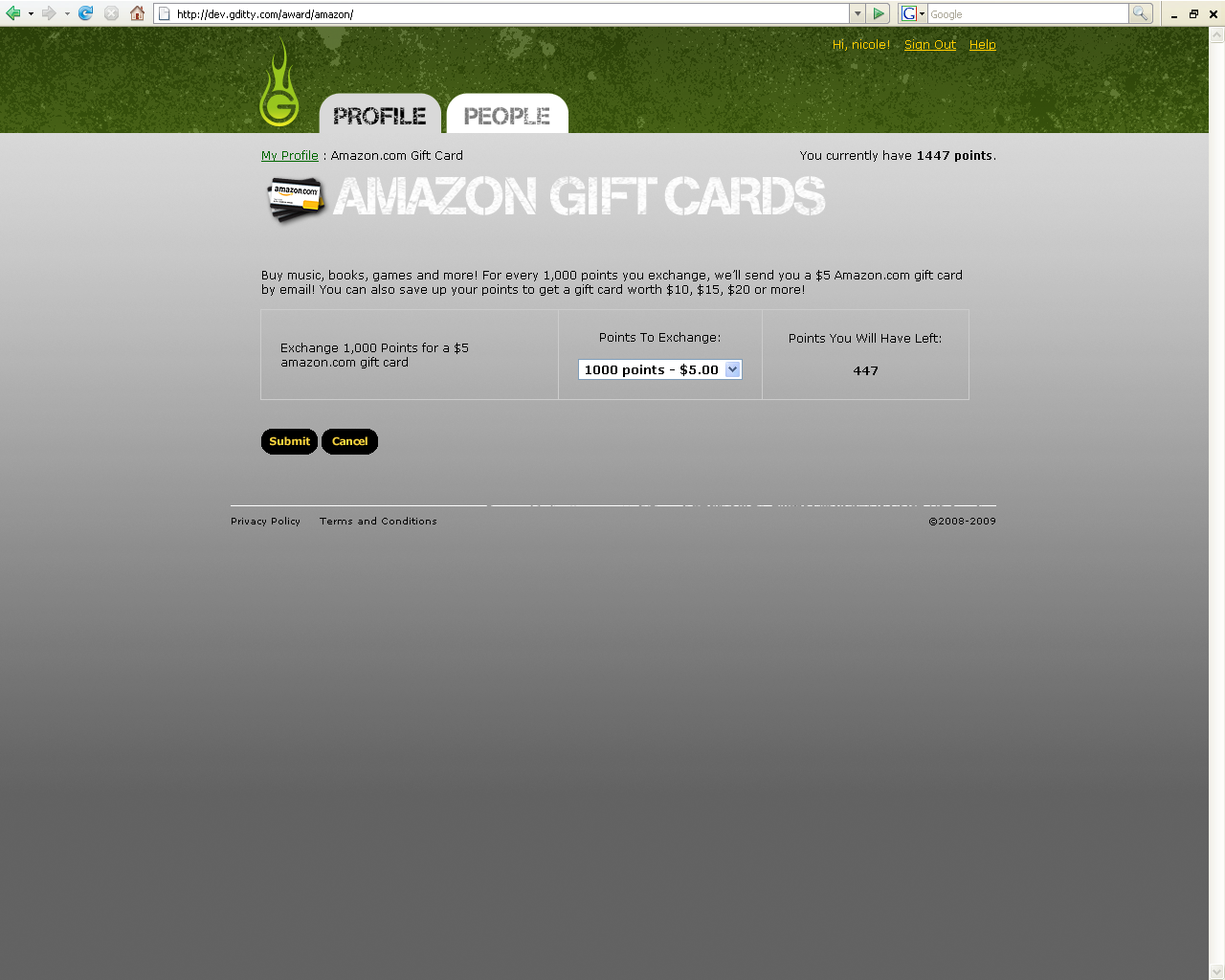


Image 10 - Individual profile page: new incentive gift card awarded


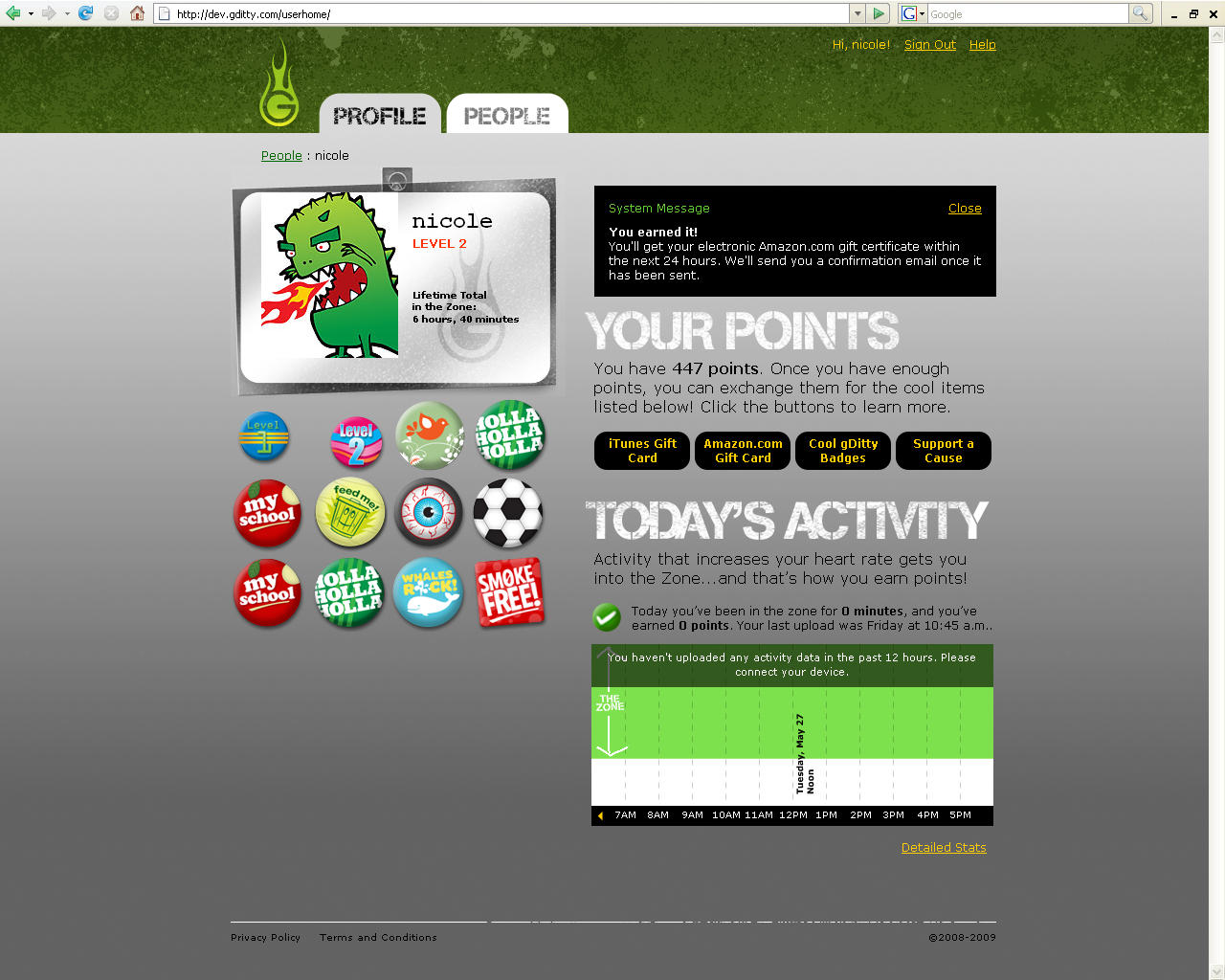


Image 11 - People page: social comparison of activity levels


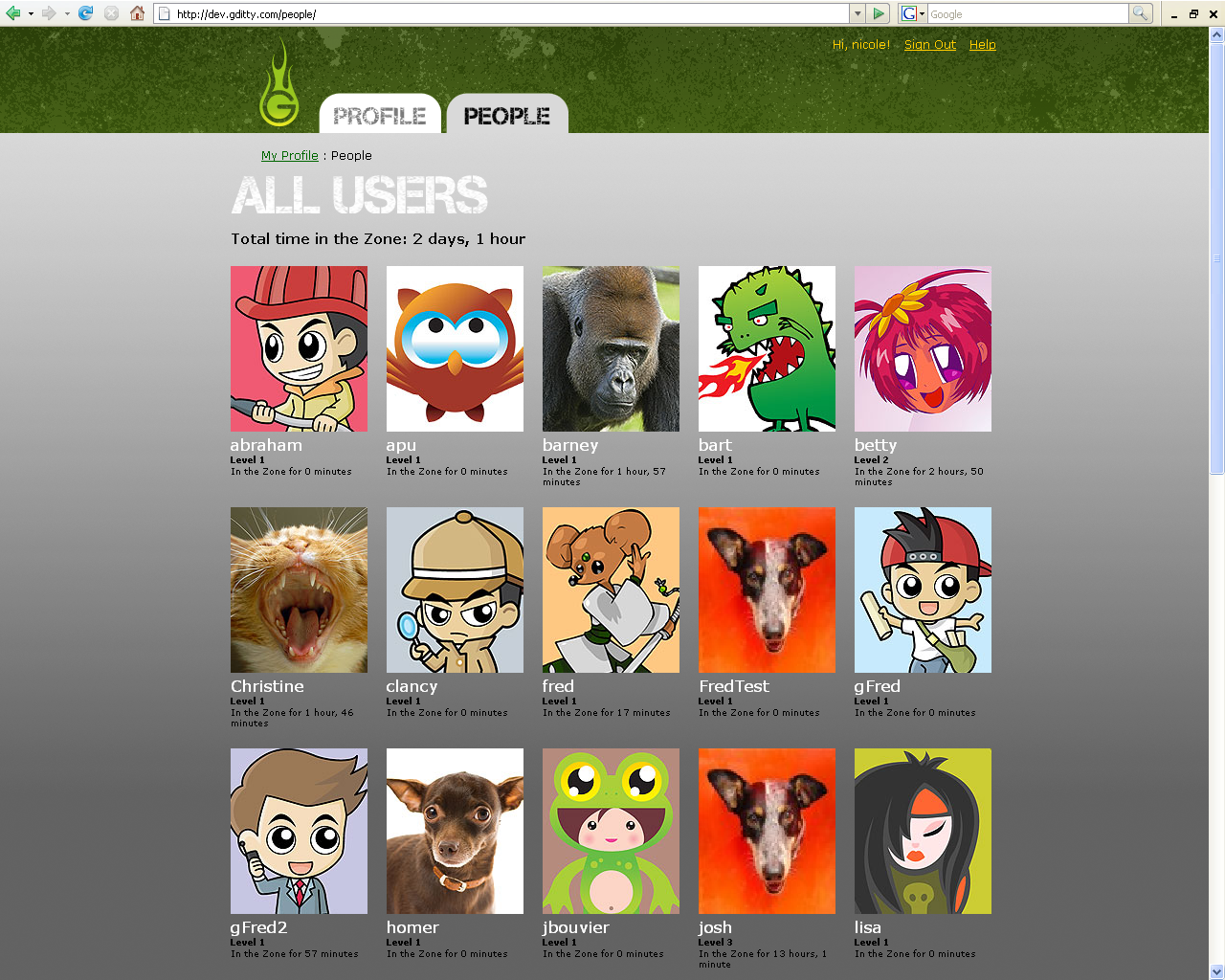

Supplement: S1 File — (DOC) [file pone.0128639.s001.doc]
